# May–Thurner Syndrome in Pregnancy-Associated Venous Thromboembolism

**DOI:** 10.1055/s-0041-1731710

**Published:** 2021-07-04

**Authors:** Mateo Porres-Aguilar, Debabrata Mukherjee

**Affiliations:** 1 Division of Hospital Medicine, Department of Internal Medicine, Texas Tech University Health Sciences Center, El Paso, Texas, United States; 2 Division of Cardiovascular Diseases, Department of Medicine, Texas Tech University Health Sciences Center at El Paso, Texas, United States

We have read the study by Jerjes-Sánchez et al with interest 1 and noted the observation that 73.8% of the 183 patients with pregnancy-associated venous thromboembolism (PA-VTE) developed predominantly left-sided lower extremity (LE) deep vein thrombosis (DVT). We would like to emphasize the potential association of pregnancy with May–Thurner syndrome (MTS), also known as iliac vein compression syndrome, described as an anatomic variant in which a compression of the thin-walled left iliofemoral vein occurred by the thick-walled right common iliac artery, resulting in intraluminal intimal hyperplasia, collagen deposition, fibrous tissue deposition, decreased venous flow, and ultimately significantly increasing the risk for extensive left-sided iliofemoral DVT. 2 3 MTS represents only 2 to 5% of all DVTs; however, retrospective autopsy and imaging studies have reported an increased prevalence between 14 and 32% in the general population 3 4 and possibly higher in pregnancy. 5 We would like to share the following questions and thoughts with the authors hereinafter.

Could you detail how many of your PA-VTE developed left-sided extensive iliofemoral DVTs?

Chan et al performed a systematic review in 124 patients with PA-DVT in which 84 (88%) of the known 96 patients, for which the side was known to be affected, were reported to have left-sided DVT; 87 (71%) of the 122 DVTs were restricted to the proximal veins, without distal calf vein involvement, and 64% (56/87) were iliofemoral DVTs, concluding that if larger studies confirmed such findings, these could positively impact our understanding of pathophysiology and derivation of diagnostic algorithms in PA-VTE. 6 Given higher proportion of left-sided LE DVTs in PA-VTE, did investigators specifically look for MTS? We believe a prior history of antenatal or postnatal MTS needs special attention, having potential future implications regarding thromboprophylaxis and duration of therapeutic antithrombotic strategies. 5

Wax et al performed a retrospective care series in four women with MTS, describing diverse clinical scenarios; previous ischemic stroke from presumed paradoxical emboli, chronic LE venous congestion treated prepregnancy with stenting, previous history of iliofemoral DVT treated with catheter-directed thrombolysis, anticoagulation and stenting, and active third trimester acute iliofemoral DVT. The first three patients received thromboprophylaxis doses of anticoagulation, while the fourth one was fully anticoagulated. 7

DeStephano et al described successful pharmacomechanical percutaneous thrombectomies in three patients with extensive iliac DVTs in pregnancy from MTS, concluding that endovascular interventions may play an important role in carefully selected patients, reducing thrombotic burden, and decreasing long-term complications like postthrombotic syndrome. 8

Regarding MTS and pregnancy, evidence suggests that MTS confers an anatomic predisposition to DVTs as vein compression leads to endothelial damage and subsequent buildup of elastin and collagen and stagnant blood flow. 9 A hypercoagulable state, such as pregnancy, compounds the risk further leading to DVTs. Since MTS is mostly subclinical and lacks symptomatology in early stages, MTS is not specifically looked for; how can we effectively put together a multidisciplinary team of experts that can appropriately and intentionally assess for MTS? How can we increase awareness and education among pregnant patients and physicians which subsequently may trigger further investigation for MTS?

Should societal clinical practice guidelines (e.g., the American College of Obstetricians and Gynecologists) consider including MTS as a strong preexisting risk factor for the development of PA-VTE? 10 Finally, what is the true impact of MTS in reproductive-aged young women for the development of PA-VTE?

Given the paucity of data regarding MTS during pregnancy and postpartum in the form of case reports and case series, 5 7 8 there is a critical need for further prospective data, to inform clinicians and researchers regarding antithrombotic and interventional/endovascular therapies for MTS, and helping make stronger shared-clinical decision-making in the complex arena of PA-VTE and MTS.
